# Barriers and Facilitators to Skin-to-Skin Contact After Caesarean Section: A Qualitative Study

**DOI:** 10.3390/healthcare14162650

**Published:** 2026-08-21

**Authors:** José Miguel Pérez-Jiménez, Miriam Fernández-Rodríguez, Thalía Flores-Alpresa, Juan Vega Escaño, Estefania Bautista Valarezo, Rocío De-Diego-Cordero

**Affiliations:** 1 Department of Nursing, Schools of Nursing, Physiotherapy and Podiatry, University of Seville, c/Avenzoar 6, 41009 Seville, Spain; jpjimenez@us.es (J.M.P.-J.); miriammfr5@gmail.com (M.F.-R.); thaliafloresalpresa@gmail.com (T.F.-A.); jvega5@us.es (J.V.E.); rdediego2@us.es (R.D.-D.-C.); 2 Human Resources and Research Unit, University Hospital Virgen Macarena, 41009 Sevilla, Spain; 3 Research Group CTS1149: Integral and Sustainable Health: Bio-Psycho-Social, Cultural, and Spiritual Approach for Human Development, 41003 Seville, Spain; 4 Department of Health Sciences, Technical University of Loja, París S/N y Praga, Loja 110104, Ecuador

**Keywords:** skin-to-skin contact, caesarean section, newborn, postpartum haemorrhage, attitudes, healthcare professionals, facilitating factors, barriers, postpartum period, family-centred care

## Abstract

**Highlights:**

**What are the main findings?**

- Skin-to-skin contact after caesarean section continues to be implemented inconsistently despite the available evidence, with structural, knowledge-related and attitudinal barriers.
- Misconceptions regarding sudden infant death syndrome and paternal skin-to-skin contact persist.

**What are the implications of the main findings?**

- These barriers are addressable through targeted and differentiated training strategies tailored to professional seniority and misconception type.
- Multidisciplinary commitment and infrastructural adaptations are key to integrating skin-to-skin contact after caesarean section into routine practice.

**Abstract:**

Background/Objectives: Despite its proven benefits, skin-to-skin contact (SSC) after caesarean section faces significant barriers to implementation. Complementing two previous randomised controlled trials conducted by our group, this descriptive qualitative study aimed to identify barriers and facilitators experienced by multidisciplinary healthcare teams, providing frontline insights into factors contributing to the inconsistent implementation of SSC. Methods: Following a phenomenological approach and the COREQ guidelines, 40 semi-structured interviews were conducted with healthcare professionals involved in caesarean care at a Spanish tertiary hospital, including anaesthetists, gynaecologists, neonatologists, midwives, nurses and nursing assistants. The data were thematically categorised at the semantic and pragmatic levels using MAXQDA software. Results: SSC implementation remains inconsistent in routine care. Despite the existence of an established institutional protocol, systematic adherence is hindered by high clinical workload, infrastructure constraints and inconsistent dissemination of guidelines. Professional barriers included gaps in training and entrenched routines, while attitudinal barriers stemmed from safety concerns following major abdominal surgery. Key facilitators included the absence of clinical complications and the positive emotional impact of SSC on family bonding. These findings provide critical diagnostic insights into implementation barriers, informing future strategies to enhance adherence to established institutional protocols. Conclusions: Although safe and feasible, post-caesarean SSC remains inconsistently implemented due to addressable barriers. Its routine adoption requires targeted multidisciplinary training, commitment from healthcare teams and infrastructural adaptations in post-anaesthesia care units (PACU).

## 1. Introduction

Early skin-to-skin contact (SSC) consists of placing the naked newborn (NB) in a prone position on the bare chest of the mother or father immediately after birth or within the first few minutes of life. Globally, caesarean sections now account for approximately 21% of all births, a proportion that continues to rise. In Andalusia, the autonomous community where the present study was conducted, the most recent available data correspond to the first half of 2025, during which public hospitals recorded a total of 20,443 births, of which 15,314 (74.9%) were vaginal deliveries and 5129 (25.1%) were caesarean sections. Yet, despite the existence of protocols and accumulated evidence of its benefits, SSC after caesarean section remains inconsistently applied across obstetric units internationally [1,2,3,4], even when the mother is awake and the newborn is healthy. Evidence from normal births indicates that mortality among preterm or low birth weight (<2.0 kg) newborns is higher when SSC is not initiated at birth [5]. Similarly, evidence from the COVID-19 pandemic indicates that separating mothers from their newborns due to this disease has negative effects [6].

The benefits of SSC have been widely demonstrated. Reported maternal benefits include a shorter delivery period, reduced postpartum haemorrhage, improved perceptions of childbirth, stronger mother-infant bonding and reduced levels of postpartum depression and anxiety. Mothers have been reported to experience less stress and greater feelings of confidence and competence in caring for their babies [2]. Improved recovery of post-caesarean plasma haemoglobin levels has also been observed [7].

In newborns, SSC favours physiological adaptation to extrauterine life, with rapid improvements in oxygenation and body temperature regulation and reduced energy expenditure [8]. SSC also increases oxytocin levels, which favour uterine contraction and colostrum ejection [1]. Higher levels of this hormone promote social interaction, bonding and attachment in mothers. Oxytocin also promotes more complex maternal behaviours by reducing anxiety, fear and pain [9,10].

Contact during the first hour of life is associated with increased expression of genes involved in the synthesis of certain molecules and hormones, such as cortisol and adrenaline, which are produced in the brain and adrenal glands of both the mother and newborn. Consequently, separation during this period may reduce the likelihood of physiological and neurobehavioural development [11]. This delay in recognising the mother may result in weaker mother–infant bonding, which can be prevented by providing a loving and nurturing environment through SSC [12]. These events fall within the concept of the Developmental Origins of Health and Disease (DOHaD), which proposes that events occurring in early life can shape long-term mental and biological development and may even contribute to the development of disease [13].

SSC is routinely practised following normal births (vaginal delivery), but remains uncommon following caesarean delivery, even when the mother is awake and the newborn is healthy and full-term. Initial contact between the mother and newborn may be delayed due to the residual effects of epidural anaesthesia or other complications. During this time, the newborn remains in a crib or, in exceptional cases, receives skin-to-skin contact with the father [2,14].

Previous studies have suggested that SSC should be initiated immediately after birth following vaginal delivery and as soon as the mother is alert and responsive following caesarean delivery. However, further evidence is needed regarding the influence of environmental and infant-related factors, support from healthcare professionals, safety concerns and constraints related to time and knowledge. In addition, further research is urgently needed into the enablers, barriers, outcomes and experiences associated with immediate SSC after caesarean section, as well as how to provide SSC safely in the operating theatre and achieve favourable short- and long-term outcomes [7].

The central question addressed by this study is therefore not whether SSC is beneficial, but why it remains inconsistently implemented following caesarean section and what institutional and professional changes are needed for it to become the standard of care.

Patient and public contributions: We thank all participants for their valuable contributions to the study.

## 2. Materials and Methods

This study builds on two randomised controlled trials, one of which was published in this journal in 2025, “Beyond Pain Management: Skin-to-Skin Contact as a Humanization Strategy in Cesarean Delivery: A Randomized Controlled Trial” (https://doi.org/10.3390/healthcare13151866), and “Does immediate skin-to-skin contact at caesarean section promote uterine contraction and recovery of the maternal blood haemoglobin levels? A randomized clinical trial” (https://doi.org/10.1002/nop2.1331). Those trials established the clinical benefits of SSC in the caesarean context. However, as documented in the wider literature [3,4], the existence of evidence does not guarantee consistent practice, as professional and organisational barriers frequently prevent the routine application of post-caesarean SSC. The qualitative study reported here was therefore designed to identify these barriers and facilitating factors among the multidisciplinary teams involved in caesarean care.

### 2.1. Research Design

A qualitative exploratory study was carried out with the aim of describing the meaning of an experience by identifying the main essential themes [15]. Semi-structured interviews were used to enable flexibility in data collection, allowing the researcher to explore how participants understand and make sense of the topic in question [16].

### 2.2. Setting and Sample

The study was carried out at a Spanish tertiary university hospital (anonymised throughout in compliance with ethical requirements; referred to hereafter as the study hospital), located in an Andalusian provincial capital. The hospital has a dedicated operating theatre area for caesarean sections adjacent to the delivery suite; postoperative recovery takes place in a separate post-anaesthesia care unit (PACU) or postpartum room. The study hospital handles more than 1700 deliveries annually, with a recent official caesarean section rate of 19.66% (approximately 1744 total births), translating to roughly 340 caesarean sections per year. The study hospital is a public tertiary referral hospital serving a catchment population of approximately 480,000 inhabitants and is affiliated with a Spanish public university as a teaching hospital for undergraduate and postgraduate health sciences education. As in most Spanish public hospitals, skin-to-skin contact following uncomplicated vaginal delivery is already routinely implemented. The barriers described in this study are specific to post-caesarean SSC. Data collection took place during the second half of 2025. The study included healthcare professionals involved in caesarean care: gynaecologists/obstetricians, midwives, anaesthetists, neonatologists/paediatricians, nurses and nursing assistants. An institutional protocol for post-caesarean SSC predated this study; however, adherence remained inconsistent. This qualitative study was therefore designed to evaluate real-practice barriers and facilitators to explain this implementation gap.

### 2.3. Procedures

Data were collected through semi-structured interviews. This approach was chosen because it combines the depth and freedom of unstructured interviews with the rigour of structured interviews, enabling participants to elaborate on topics of concern while allowing the researcher to seek clarification and explore emerging themes. Purposive sampling was used. Interviews were conducted by two members of the research team experienced in qualitative methodology: a research technician and a nurse researcher. To mitigate potential social desirability bias or power dynamics associated with the principal investigator’s clinical and supervisory position, full anonymity and confidentiality were explicitly guaranteed prior to each session, and a standardized semi-structured interview guide was followed. Thematic saturation was assessed iteratively, and recruitment was stopped after 40 interviews when no new codes emerged from the preceding three interviews. Furthermore, to ensure analytical neutrality, data analysis and the coding process were cross-examined through continuous reflexivity and investigator triangulation with the rest of the research team.

### 2.4. Recruitment and Selection

Of the 52 professionals approached, 40 agreed to participate (response rate, 76.9%). Eight declined due to lack of time or interest and four were excluded due to scheduling conflicts. All six targeted professional groups were represented in the final sample (anaesthetists, gynaecologists/obstetricians, neonatologists, midwives, nurses and nursing assistants). Seniority varied across professional groups. Of the 8 anaesthetists interviewed, 4 were residents in training; of the 4 neonatologists interviewed, 3 were residents in training and 1 was a senior clinician. By contrast, all 6 gynaecologists/obstetricians and all 8 midwives held permanent or casual staff positions. This imbalance in seniority, particularly the limited representation of senior neonatologists, should be considered when interpreting differences between professional groups and may itself account for some of the divergence reported between groups. Participation was entirely voluntary. To protect participants from any perceived coercive pressure, particularly residents and temporary staff, it was made clear that non-participation would have no consequences for employment or training assessments. Recruitment was managed independently by the research team rather. One hundred percent of the transcripts were independently coded by two researchers. Discrepancies arising during the process were formally documented and resolved through discussion and consensus during regular meetings, establishing a final coding framework that was subsequently validated by a third member of the research team. Interviews were scheduled at 9:00 am, 9:30 am, 10:00 am and 10:20 am to coincide with staff changeovers and minimise disruption to clinical duties, with 4 interviews conducted per day. Participants chose the interview location themselves, ensuring privacy and freedom from interruption. Interviews lasted approximately 30 min, with additional time offered as needed.

Prior to the start of the interview, participants were asked to read the informed consent form, which outlined the purpose of the study, risks, benefits, costs, confidentiality and requirements, as well as providing the researcher’s contact details for any questions that might arise.

### 2.5. Instrument

An interview guide consisting of open-ended questions divided into two sections was used. The first section covered sociodemographic characteristics, while the second addressed the aspects indicated above. This interview guide is presented in Table 1.

### 2.6. Data Analysis

**Figure 1:**
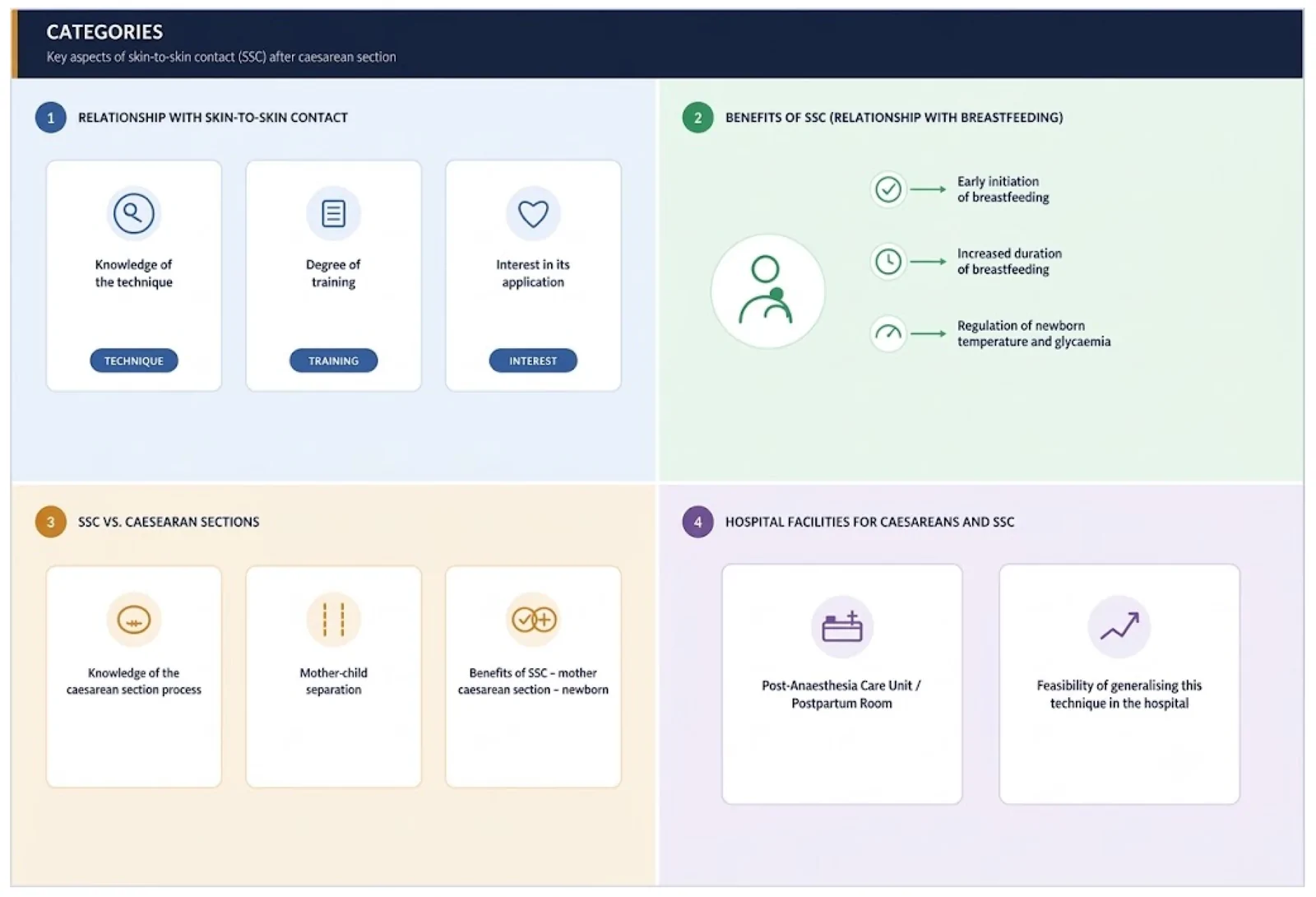
Categories.

Thematic analysis followed a systematic coding process. Initially, a preliminary codebook was developed deductively from the research questions and was subsequently expanded inductively as new categories emerged during the analysis, in line with the circular principle of qualitative inquiry. Coding was performed independently by two members of the research team using MAXQDA 2022. Discrepancies were resolved through discussion until consensus was reached. No formal inter-rater reliability statistic was calculated, as consensus-based methods are standard practice in phenomenological research. The final coding framework encompassed four overarching categories (Figure 1): relationship with SSC (knowledge of the technique, degree of training, interest in its application); benefits of SSC (relationship with breastfeeding); SSC vs caesarean sections (knowledge of the caesarean section process, mother-infant separation, benefits of SSC for the mother and newborn following caesarean section); and hospital facilities for caesarean sections and SSC (PACU, feasibility of generalising this technique within the hospital).

Qualitative analysis was carried out using the following steps: (1) familiarisation with the data; (2) generation of categories; (3–5) searching, reviewing and defining themes; and (6) preparation of the final report, in which participants were identified by participant number, gender, age and place of work. Transcription, literal reading and manual theoretical categorisation were also performed. The categories were analysed at the semantic and pragmatic levels. Semantic analysis involved examining the meanings that participants attributed to the topics or categories of interest. The categorised text fragments were then interpreted in relation to their context, thereby reaching the pragmatic level of analysis.

Furthermore, MAXQDA 2022 qualitative data analysis software was used for data analysis and management. MAXQDA is a specialised software application designed for qualitative, quantitative and mixed methods research. Negative case analysis was undertaken as an integral part of the coding process. Statements that diverged from the emerging themes or expressed a different degree of agreement with the majority view were flagged and retained rather than discarded and were used to refine and qualify the final categories. This was particularly relevant because participants varied noticeably in their degree of conviction regarding SSC after caesarean section. Several expressed strong, unqualified support, while others expressed conditional agreement or scepticism related to workload, training or perceived risk. These divergent accounts are reported explicitly, rather than being subsumed into the dominant narrative, in the age-related divergence described in Section 4.1.

### 2.7. Trustworthiness

Trustworthiness was addressed across four dimensions. Credibility was supported by data triangulation between the literature and the qualitative findings, member checking with selected participants and the use of verbatim quotations in the Results. Transferability was facilitated by the detailed description of the sample and setting provided in this report. Dependability and confirmability, established criteria in qualitative research [15], were enhanced through an independent dual-coding process and by following the Consolidated Criteria for Reporting Qualitative Research (COREQ) checklist. The completed COREQ checklist is provided as Supplementary Materials. Regarding researcher reflexivity: the principal investigator holds a clinical and managerial role at the study hospital and has published two randomised controlled trials on SSC in caesarean sections. This dual role may have predisposed some participants to provide socially desirable responses. To reduce this risk, the interviews were introduced as an exploration of professional experience rather than an evaluation of SSC practice, and participants were explicitly informed that sceptical or negative views were equally valid. MAXQDA was used for qualitative data analysis and management, and the study followed the COREQ guidelines [17].

### 2.8. Ethical Considerations

This study followed the ethical principles for medical research involving human subjects set out in the World Medical Association Declaration of Helsinki and its subsequent revisions in Tokyo (1975), Venice (1983), Hong Kong (1989), Somerset West (1996), Edinburgh (2000), Washington (2002) and Seoul (2008), as well as the Declaration of Fortaleza (Brazil) and the Oviedo Convention.

Participation in the study was voluntary. The invitation to participate was presented as a healthcare research proposal, and participants were informed of the study objectives, procedures, potential risks, confidentiality and their right to withdraw consent at any time without explanation or consequence. Prior to each interview, participants were provided with a written participant information sheet and gave written informed consent. Signed consent forms were retained securely by the principal investigator. Participants were identified only by an assigned case number; no personal identifiers appeared in the data or in this report. The study complies with Spanish data protection legislation (Organic Law 3/2018 of 5 December on Personal Data Protection and Guarantee of Digital Rights). Ethical approval was granted by the Andalusian Research Ethics Committee (internal code: 0428-N-17).

## 3. Results

This section presents the results of the detailed analysis of the 40 interviews conducted during the study. First, the characteristics of the participants are presented (Table 2). The results are then presented according to the established thematic categories. Where relevant, differences in perspectives between professional groups are highlighted, and barriers are classified as structural (staffing, infrastructure and physical space), knowledge-based (training deficits and unfamiliarity with the evidence) or attitudinal (resistance rooted in routine, safety concerns or low personal motivation).

### 3.1. Participant Profile

The interviewees were healthcare personnel involved in the care of women undergoing caesarean section, including anaesthetists, gynaecologists, neonatologists, midwives, nurses and nursing assistants.

### 3.2. Knowledge and Training Regarding SSC

Regarding the level of knowledge about SSC, all participants were familiar with the technique, although their knowledge did not cover all aspects. Most interviewees referred to the definition of the process, the placement of the newborn on the mother’s chest at birth and some of its benefits. Not all participants mentioned the safety measures that should be followed or the time required for SSC to be effective. They also did not mention when newborn identification procedures or the administration of prophylactic medication should be carried out. Few highlighted that SSC is sometimes performed by the father.

“*…a little bit… I think it favours the early care of the newborn, the relationship with the parents and reduces anxiety.*” (Man, 50 years old, healthcare assistant [HCA]).

“*…in my opinion, it is about avoiding separation between the mother and baby immediately after birth.*” (Woman, 63 years old, midwife).

“*…when the child is born, they are placed on the mother’s chest and remain there for several hours.*” (Man, 42 years old, nurse).

Some professionals had working knowledge of the procedure but expressed concerns that women might be uncomfortable or that SSC was too brief to be meaningful in practice.

“*…basically, the centre focuses on having the child at the breast. I attended a course where the mothers acted as an incubator*.” (Woman, 58 years old, nurse).

“*…there are women who should not be pressured because they may end up rejecting the baby, so we have to be careful.*” (Woman, 51 years old, gynaecologist/obstetrician).

“*…at first, it’s a procedure that makes women happy, but it only lasts a short time, about five or ten minutes, because the paediatrician comes by, at least in my experience.*” (Woman, 64 years old, midwife).

In contrast, almost all professionals reported that they had not received training in this procedure. This finding is important to consider, as training is closely related to professionals’ interest in and acceptance of performing the technique.

“*…well… I have never been told about these things. I only know what I have read in manuals.*” (Woman, 29 years old, anaesthetist).

“*I haven’t received any information. I became aware of the issue on my own initiative by reviewing the protocols of some departments, although it is implemented in very few of them.*” (Woman, 38 years old, midwife).

“*…provided or promoted by the institution, none.*” (Man, 28 years old, resident physician, anaesthesia).

### 3.3. Acceptance and Interest in Its Application

To assess the level of personal interest in this technique, interviewees were asked about their interest in the topic. Most reported a moderate level of interest. Their acceptance of the practice was influenced by concerns about ensuring that both the mother and newborn met the necessary conditions for SSC to be performed safely. Participants expressed uncertainty about their ability to provide the appropriate care for both the mother and newborn. One issue emerged repeatedly throughout the interviews and was present in almost all responses; namely the lack of sufficient staff to perform SSC safely. Another issue raised in almost all responses was the lack of appropriate hospital facilities.

“*… it seems to me an advisable technique as long as maternal or foetal conditions do not prevent it*.” (Woman, 47 years old, gynaecologist/obstetrician).

“*I think it’s essential whenever possible, since it encourages early feeding and the mother-child relationship.*” (Woman, 54 years old, gynaecologist/obstetrician).

“*…honestly… the subject doesn’t interest me very much because I believe that newborns are better in the crib, as they don’t lose heat, and if they need to be resuscitated, it is easier. Also, after so many hours in the operating theatre, the mother may not feel like holding the baby.*” (Woman, 61 years old, midwife).

### 3.4. Benefits of SSC

The professionals were also asked about their views on the benefits of SSC for the mother and newborn. All interviewees agreed that SSC was important for the mother, newborn and family as a whole, although some expressed reservations.

“*…scientifically, no one has shown me the importance of SSC with the father, but personally, with the body hair, sweat… it doesn’t seem right to me.*” (Woman, 63 years old, midwife).

“*…the possible relationship between skin-to-skin contact and an increased risk of sudden infant death is being investigated.*” (Woman, 54 years old, gynaecologist/obstetrician).

These two responses are particularly noteworthy. The first reflects a personal aversion to paternal SSC that is not supported by the available evidence. The literature indicates that paternal SSC is safe and beneficial, with reported benefits for neonatal thermoregulation, cardiorespiratory stability and parental bonding [1]. The second reflects a misconception regarding sudden infant death syndrome (SIDS). Current evidence does not support an association between SSC performed under clinical supervision and an increased risk of SIDS. On the contrary, SSC has been associated with reduced neonatal stress and greater stability of vital signs [18]. Both responses highlight gaps in evidence-based training that could be addressed through targeted education.

“*Well… in my view, it’s a feeling that can’t be explained. It’s very emotional. That skin… that body… your baby. It’s such an intimate moment, your own flesh and blood*” (Woman, 53 years old, HCA).

“*Of course…this is the most important moment for the woman. It’s the first time you feel your child against your skin after nine months. It’s a miracle*” (Woman, 61 years old, nurse).

### 3.5. Relationship with Breastfeeding

To explore the perceived relationship between SSC and breastfeeding, all participants were asked about this association. As in other categories, a minority did not recognise the relationship or expressed doubts, but overall there was a high level of agreement. The large majority of participants reported a positive relationship, highlighting that the earlier SSC was initiated, the sooner breastfeeding was established.

“*It seems essential to me whenever possible because promoting early feeding also favours the sucking reflex and helps the milk come in.*” (Woman, 29 years old, anaesthetist).

“*… SSC can favour the establishment and duration of breastfeeding.*” (Woman, 42 years old, nurse).

“*…yes… because early skin-to-skin contact helps establish breastfeeding. Without it, breastfeeding can be more difficult.*” (Woman, 25 years old, resident physician, neonatology).

### 3.6. Skin to Skin Contact After Caesarean Sections

The professionals were then asked whether their views were the same in the context of caesarean section. Regarding their knowledge of the caesarean section process and mother-infant separation, at this hospital, once the procedure was completed, women who had undergone a caesarean section sometimes followed a different pathway from their newborn. The mother was transferred to the PACU, while the newborn was taken to a room with the father. Interviewees were first asked whether they were aware of this process. Analysis of the responses showed that all professionals were aware that the mother was sometimes transferred to the PACU while the newborn was taken to a room with the father.

“*Yes… children and mothers are separated while the effects of anaesthesia wear off.*” (Woman, 58 years old, nurse).

“*In the operating theatre, I have never seen the newborn placed on the father’s chest. In fact, the mother and newborn are separated for at least two hours.*” (Woman, 64 years old, midwife).

“*…there is no skin-to-skin contact… the mother and newborn are separated.*” (Woman, 27 years old, gynaecologist/obstetrician).

Alongside the above, we explored attitudes towards SSC specifically in the context of caesarean section. The most frequently expressed view was that professionals did not consider SSC beneficial in this setting, citing the fact that caesarean section is major surgery, maternal discomfort and the belief that its appropriateness should be determined by the attending professionals. Some staff members stressed that prior professional authorisation was required. There was broad agreement that SSC following vaginal delivery was beneficial, but this view was not shared in relation to caesarean section.

“*No, I don’t think it is beneficial for either of them, neither the mother nor the newborn. I think that immediately after major surgery such as a caesarean section, skin-to-skin contact is not beneficial. What’s more, with increasing information and social pressure surrounding SSC, many women may feel pressured into it. Despite being in pain and unable to care for the newborn, they may feel obliged to attempt SSC.*” (Woman, 47 years old, gynaecologist/obstetrician).

“*I imagine so, although you would have to ask the mother, as it depends on how tired she is. Some women can be quite distressed afterwards.*” (Man, 29 years old, resident physician, anaesthesia).

“*It could be... although there may be greater risks following a caesarean section than after a vaginal delivery. As long as the mother does not experience complications such as bleeding, it may be possible, but for this very reason, I don’t know to what extent SSC can be beneficial for a woman who has just undergone a caesarean section.*” (Woman, 64 years old, midwife).

These responses reveal two recurring beliefs that are not supported by the evidence already established at this hospital. The first is that caesarean section is inherently too dangerous for SSC to be attempted, while the second is that encouraging SSC after caesarean section amounts to coercing women who are not well enough to participate. Two randomised controlled trials conducted at this hospital have already demonstrated that immediate SSC after caesarean section without identified safety concerns and improves maternal recovery when appropriate clinical criteria are applied and the woman’s consent is respected throughout [7,9]. The perceptions of danger and coercion described here therefore appear to reflect gaps in training and communication rather than established clinical contraindications, reinforcing the need for structured, evidence-based training identified elsewhere in this study.

### 3.7. Hospital Infrastructure and Staffing Barriers

During the interviews, participants were asked whether they considered the hospital infrastructure suitable for carrying out SSC safely. They were also asked about their general perception of the organisation of space in the gynaecological areas and the PACU and to describe their views in more detail. Finally, participants were asked whether they considered the hospital’s current infrastructure adequate for extending SSC to all mothers following caesarean section and whether their attitude toward the practice, whether proactive or otherwise, would remain the same if SSC were routinely implemented at the hospital where they worked.

Disagreement was relatively widespread. One of the most frequently cited reasons for participants’ reservations about SSC following caesarean section was that the hospital infrastructure was not adequately equipped to support the practice. Similarly, participants repeatedly noted that staff were insufficiently prepared and had not received the necessary training.

“*No…there are still no trained staff to do this.*” (Man, 61 years old, nurse).

“*No… there isn’t the infrastructure or the capacity… everything falls to the midwife… the newborn, the mother, everything… I’m not a paediatrician… I can’t care for the newborn if something happens…*” (Woman, 59 years old, gynaecologist/obstetrician).

Most participants did not support the routine implementation of SSC due to insufficient staffing and the lack of an appropriately equipped space in which to care for the mother and newborn together.

“*A series of adaptations could be made… but at present, the necessary conditions are not in place.*” (Woman, 56 years old, nurse).

“*Everything depends on what we want to achieve…even just with a bed…it comes down to willingness.*” (Man, 28 years old, resident physician, anaesthesia).

“*They don’t exist… because after a caesarean section… the mother and newborn should not have to remain separated until she is discharged from the recovery room [PACU].*” (Man, 50 years old, neonatologist).

In addition, participants were asked whether they considered the PACU, to which mothers are sometimes transferred following caesarean section rather than being taken directly to the designated postpartum rooms, an appropriate setting in which to care for the mother and newborn together and facilitate SSC.

All interviewees except one considered this space unsuitable for this purpose. The main concerns related to discomfort, a lack of safety for the mother and newborn and, in particular, insufficient staffing.

“*…no…there are other patients with multiple pathologies…some of which, for example, could be infectious…so we would be exposing the newborn. In addition, the temperature is usually lower, so the newborn could become cold…and then there are other patients recovering from surgery who need peace and quiet. With a newborn crying… it’s difficult.*” (Man, 50 years old, neonatologist).

“*It isn’t the most appropriate place… it’s shared with other patients and the environment isn’t ideal.*” (Man, 42 years old, nurse).

“*Anything is possible if you really want to make it happen…if there is goodwill and a willingness to make it work…*” (Woman, 47 years old, gynaecologist/obstetrician).

“*[If we were to set it all up for them…] who is going to look after these women and the newborn? Because I can’t do it all… not everything can fall on us.*” (Woman, 61 years old, midwife).

“*I totally agree… as long as it’s properly organised… they would need to set up a dedicated space in the recovery room [PACU].*” (Woman, 27 years old, gynaecologist/obstetrician).

“*I would agree… as long as the appropriate hygiene measures were in place… so that skin-to-skin contact was safe and did not pose a risk to the mother or newborn… especially the newborn.*” (Woman, 61 years old, nurse).

“*…strongly agree…but only if the appropriate conditions were in place …with adequate postoperative monitoring…then I would agree.*” (Man, 50 years old, neonatologist).

There was no general agreement among healthcare professionals that SSC following elective caesarean section was beneficial for the mother and newborn. Some participants referred to established routines, expressing views such as “*why should it change now?*” and “*this technique has never been carried out and has not been tested*”.

A lack of training, staff shortages and inadequate hospital infrastructure were the most frequently cited reasons for participants’ reluctance to support this procedure at the hospital where they worked.

## 4. Discussion

The aim of this study was to identify the barriers and enabling factors associated with the implementation of SSC following caesarean section. The main findings indicate that limited training among healthcare professionals, the need to adapt available hospital spaces, safety concerns and staff shortages are the principal barriers to implementing this practice. By contrast, the absence of clinical complications and the positive experience of SSC for families emerged as important facilitators of its implementation.

The two favourable elements identified in this study, namely the absence of clinical complications and the perceived benefits of SSC for families, were not sufficient on their own to overcome the structural and training-related barriers described above. Rather, they appeared to operate primarily at the attitudinal level, reinforcing individual clinicians’ willingness to support SSC when the opportunity arose, without substituting for the institutional investment required to address training gaps or adapt PACU infrastructure. This suggests that these two types of factors operate at different levels of the same implementation problem. Positive attitudes alone are unlikely to translate into routine practice without corresponding structural and educational change.

### 4.1. Knowledge, Training and Attitudes Towards Skin-to-Skin Contact

The health professionals interviewed described SSC in varied and, at times, inaccurate terms, reflecting gaps in their knowledge that are consistent with findings reported by other authors [3]. This finding also supports previous research highlighting the need to assess healthcare professionals’ knowledge of SSC and its application in obstetric operating theatres during caesarean delivery [19,20].

This knowledge gap, together with the disruption that SSC may introduce to established clinical routines, emerged in the present study as a key barrier to its early implementation following caesarean delivery. The literature further highlights the importance of considering several factors when implementing SSC in healthcare settings, including the clinical environment, newborn-related factors, support for healthcare professionals, safety concerns and limitations in time and knowledge [21].

A notable finding was the presence of misconceptions among a subset of professionals. Some participants associated supervised SSC with an increased risk of sudden infant death, while others expressed concerns about the risk of neonatal hypothermia. However, the available evidence does not appear to support these concerns. Supervised SSC in the operating theatre and PACU has been associated with improved neonatal thermoregulation and cardiorespiratory stability and reduced stress hormone levels, rather than with increased risk [2,18]. Similarly, concerns regarding paternal SSC do not appear to be substantiated in the literature reviewed. These findings suggest that structured, profession-specific training programmes extending beyond generic SSC education may be beneficial. This pattern is broadly consistent with international literature from North America, Australia, and East Asia, where training deficits and safety concerns not clearly supported by evidence have frequently been identified as barriers to the implementation of SSC following caesarean section [1,22,23].

When the data were examined by age group, a clear generational divergence emerged regarding resistance to SSC following caesarean section. Professionals aged 60 years or older appeared noticeably more likely than their younger colleagues to express resistance, frequently relying on entrenched clinical routines and questioning the necessity of modifying long-established practices (e.g., ‘why should it change now?’). For these senior clinicians, resistance appeared to be rooted in years of habituation to traditional surgical workflows and conservative risk perceptions surrounding postoperative care. Conversely, younger professionals and residents expressed greater openness and interest in implementing early SSC, perceiving it as a natural evolution towards humanised maternity care. However, their ability to drive change was often constrained by hierarchical dynamics and the lack of explicit institutional endorsement, leaving them hesitant to challenge established unit practices. Expanding the understanding of this generational gap is essential: addressing resistance in senior staff requires targeted unlearning strategies and updating evidence-based risk perceptions, whereas supporting junior staff requires formal institutional protocols and leadership empowerment to bridge the gap between positive attitudes and actual clinical practice.

### 4.2. SSC Benefits

The women who participated in the two randomised controlled trials preceding this qualitative study reported very high levels of comfort and satisfaction with SSC following caesarean section [7,9]. However, other authors have reported that SSC may cause stress in some mothers who do not feel able to engage in the practice during the postoperative period [24]. Similar concerns were expressed by some of the professionals interviewed in the present study.

Regarding factors that facilitate SSC, professionals highlighted the perceived benefits of early SSC for the newborn, as well as the sense of well-being, support and security experienced by mothers when holding their newborn skin-to-skin. They also emphasised the positive experience for parents of being involved in their newborn’s care from the earliest stages. Avoiding unnecessary separation and taking advantage of the newborn’s early alert period may facilitate SSC [1]. Previous studies have also reported that early SSC can improve mothers’ perceptions of the birth experience and promote mother-infant bonding [23].

Another facilitating factor identified was the absence of complications in both the mother and the newborn. Indeed, some authors have described SSC as an indicator of good clinical practice and have associated its absence with less favourable maternal and neonatal outcomes [2].

Given the importance of SSC in promoting mother-infant bonding, previous studies have highlighted the need to offer immediate or early SSC to all eligible mothers following caesarean section. Immediate or early SSC has been associated with several maternal and neonatal benefits, including enhanced well-being, increased interaction between parents and the newborn, reduced maternal pain and anxiety, improved physiological stability in both the mother and the newborn and better breastfeeding outcomes [25].

### 4.3. Hospital Infrastructure and Facilities for SSC Following Caesarean Section

Another identified barrier relates to concerns about the physical environment in which SSC is performed. Participating midwives highlighted the lack of precedent for performing SSC within the operating theatre in this manner, expressing concerns about clinical safety and the attribution of professional liability should a maternal or neonatal adverse event occur. Similarly, healthcare assistants working in the operating theatre expressed concerns about the risk of neonatal hypothermia during SSC on the mother’s chest, noting that the practice departed from established clinical routines. By contrast, participants from neonatology, operating theatre nursing and the PACU generally considered SSC during maternal recovery to be feasible, provided that dedicated and appropriately adapted space was available. Finally, some participating gynaecologists cited concerns about neonatal safety and maternal discomfort following major surgery, particularly regarding the stability and clinical monitoring of the newborn in this setting [1].

Among the structural barriers, participants highlighted the need to adapt the physical environment and designated hospital spaces to support SSC, as well as to provide suitable accommodation for the birth companion during maternal recovery. Appropriate planning is essential to ensure that SSC can be performed safely. In this regard, previous research has shown that operational barriers within the operating theatre can be successfully addressed during caesarean delivery, facilitating the initiation of breastfeeding within 30 min of birth [1]. It should be emphasised that, in the majority of caesarean procedures, SSC is feasible both in the operating theatre and in the PACU, provided that maternal and neonatal clinical status allows for it. Although immediate postoperative separation has historically been justified on the grounds of neonatal safety and reducing the risk of complications, evidence suggests that this risk is low in uncomplicated elective or urgent caesarean sections, supporting the routine promotion of early SSC to enhance the humanisation of surgical birth [22].

### 4.4. Feasibility of Implementing SSC Across the Hospital

Regarding the feasibility of implementing SSC across the hospital, one of the primary barriers identified by participants was staff shortages, consistent with previous studies citing insufficient staffing and time constraints. Midwives, in particular, emphasised that limitations in staffing, time and physical space hinder the routine implementation of SSC [26].

According to the findings of this study, successful implementation of SSC requires the establishment of multidisciplinary committees involving professionals who participate in caesarean sections, in order to establish consistent criteria and develop clinical protocols [8]. Appropriately trained professionals are also essential. In this regard, previous studies have identified a lack of training among key healthcare professionals as a barrier to the implementation of SSC. Collaboration among all healthcare professionals is essential for implementing SSC in the operating theatre, highlighting the need for additional staff with specific knowledge and training in the practice [27]. In addition, staff shortages have been identified by several authors as a barrier to the implementation of early SSC [1].

Another barrier to the implementation of SSC was participants’ concern about sudden infant death and the risk of neonatal hypothermia. Healthcare professionals also expressed concerns about their ability to monitor the clinical condition of both the mother and newborn, particularly during the first hours following caesarean section, due to the perceived risk of apnoea. However, other authors have reported multiple benefits of SSC for the newborn, including improved thermoregulation and cardiorespiratory stability, reduced stress after birth and facilitation of the transition to extrauterine life [18].

Strengths and limitations

The main limitations of this study are its single-centre design and the purposive sampling strategy, which, while appropriate for qualitative research, limit transferability to hospitals with different organisational structures or staffing models. A further limitation concerns the transferability of these findings across caesarean-section subtypes. Participants were not systematically asked to distinguish between their experiences of facilitating SSC following elective and emergency procedures, and the barriers identified may not apply equally to both. Emergency caesarean sections are typically performed under greater time pressure, more frequently involve general rather than neuraxial anaesthesia and allow less opportunity for antenatal preparation of the mother and family for SSC. The organisational and training barriers identified in this study may therefore be more pronounced in the emergency setting. Elective caesarean sections, planned in advance and generally performed under neuraxial anaesthesia with the mother awake, may offer a more readily transferable model for the implementation of early SSC. Future studies should examine these two contexts separately, both in the study hospital and in other settings. Data collection was also more demanding than initially anticipated, as securing participation required the prior agreement of each professional approached. The absence of patients’ perspectives is a further limitation; future research should incorporate the experiences of women who have undergone caesarean section to complement the staff perspectives captured here.

The principal strengths are the breadth of the multidisciplinary sample, spanning multiple professional groups involved in caesarean care, the independent dual-coding process and the rigorous application of the COREQ checklist. This study forms part of a programme of research comprising three complementary papers: two randomised controlled trials establishing the clinical benefits of SSC following caesarean section [7,9] and the present qualitative study exploring the translation of this evidence into practice. This progression from evidence generation to implementation is a key strength of the research programme as a whole.

Recommendations and implications for practice and further research

It is important that obstetric units establish a structured, multidisciplinary approach to implementing SSC following caesarean section. Training should be systematic and profession-specific, as anaesthetists, midwives, neonatologists and PACU nurses encounter distinct barriers and may require tailored education. The PACU should be appropriately adapted to enable safe, supervised SSC alongside the care of other postoperative patients. Patient-centred information for expectant mothers should be integrated into antenatal care so that SSC following caesarean section can be considered as part of birth planning. Future research should evaluate the clinical outcomes of SSC programmes once implemented, particularly rates of postpartum haemorrhage, breastfeeding initiation and maternal anxiety, and should incorporate the perspectives of women themselves.

Given finite institutional resources, prioritisation should reflect the frequency of the barriers identified and the resources required to address them. Training deficits and safety misconceptions, which were among the most frequently reported barriers and may require fewer resources to address, could be prioritised through profession-specific training. Adaptation of the PACU infrastructure, which would require greater institutional investment, could then follow once staff are appropriately prepared. A pilot intervention limited to the PACU and the surgical critical care unit could combine structured training with minor space reorganisation and be evaluated over a defined period (e.g., six months) using measurable outcomes. These could include the proportion of caesarean sections in which SSC is initiated within 30 min, breastfeeding initiation rates and staff-reported confidence. Such an intervention would allow the hospital to assess feasibility before wider implementation and generate the outcome data identified as necessary above.

Relevance to clinical practice

In many clinical settings, immediate SSC is not routinely performed following caesarean section. The absence of early SSC may adversely affect the recovery of uterine contractility and may be associated with an increased risk of postpartum haemorrhage.

Caesarean section rates are increasing exponentially and currently account for 21% of all births. Postpartum haemorrhage remains an important cause of maternal mortality, accounting for 35% of maternal deaths. Women undergoing caesarean section may be at a greater risk of postpartum haemorrhage because of factors related to abdominal surgery and lower circulating oxytocin levels, a hormone that promotes uterine contractility.

To date, most published research on SSC has focused on vaginal births or other birth contexts. It is therefore important to understand the level of acceptance and knowledge of SSC among professionals involved in caesarean delivery, in order to identify the conditions needed to support effective mother-infant bonding following caesarean section.

By examining professionals’ attitudes and levels of training from a phenomenological perspective, our study contributes scientific knowledge that can inform the design and implementation of SSC following caesarean section.

SSC may contribute to greater maternal stability by reducing the risk of postpartum haemorrhage, as well as to improved neonatal adaptation by enabling the newborn to remain close to the mother.

Keeping mothers and newborns together following caesarean delivery promotes family-centred care and may increase satisfaction among women, nurses and obstetric staff. Extending SSC across healthcare settings may not require substantial additional resources and, based on the evidence reviewed, could have a meaningful impact on the child’s physical and psychosocial development. However, dedicated cost-effectiveness research is needed to confirm this.

## 5. Conclusions

This study identifies three interrelated barriers to the routine implementation of SSC following caesarean section: gaps in knowledge and training among healthcare professionals, attitudinal resistance associated with established routines and safety concerns regarding the postoperative setting, and logistical and spatial constraints alongside staffing pressures. These barriers are mutually reinforcing: professionals with limited training may feel less confident, institutions without clear protocols provide little guidance for practice and understaffed units face challenges in safely supervising SSC. These barriers should therefore be addressed simultaneously.

In addition, the absence of complications associated with SSC following caesarean section, its perceived benefits for families and the sense of attachment, comfort and confidence experienced by mothers were identified as facilitating factors for its implementation.

These findings can be interpreted within the Consolidated Framework for Implementation Research (CFIR) [28,29]. Viewed through this framework, the knowledge deficit falls within the *Characteristics of Individuals* domain, while attitudinal resistance rooted in established routines, together with limitations in space and staffing, falls within the *Inner Setting*, reflecting organisational culture and resource availability. By contrast, the absence of complications and the technical simplicity of the procedure align with *Intervention Characteristics*, supporting its perceived compatibility with clinical workflows. The Theoretical Domains Framework (TDF) provides a complementary, more granular analysis of individual-level factors, highlighting how knowledge gaps, erroneous beliefs about consequences and role ambiguity may shape clinical decision-making.

Although healthcare professionals currently perceive SSC as more readily achievable following vaginal delivery, these findings suggest that their concerns about its implementation following caesarean section may be primarily organisational rather than purely clinical. When appropriate professional training is provided and suitable PACU infrastructure is in place, SSC following caesarean section can be implemented safely and is generally viewed positively.

## Figures and Tables

**Table 1:** Interview Guide. Semi-structured interview guide on skin-to-skin contact (SSC) after caesarean section.

No.	Interview Question
1	Could you tell me what you know about skin-to-skin contact with the newborn?
2	Could you tell me a little about your interest in this technique?
3	Could you tell me about the level of training you have received in this procedure?
4	Do you think it provides benefits for the mother and newborn? Please explain why.
5	Do you think that skin-to-skin contact immediately after birth is important for establishing breastfeeding?
6	Do you know what happens in cases of caesarean section?
7	After a caesarean section, do you consider it beneficial for the mother to have skin-to-skin contact with the newborn within the first half hour? Do you consider it beneficial for the newborn?
8	Do you think that the hospital where you work has the appropriate resources and facilities to carry out this practice? Please explain.
9	Do you consider the PACU adequately prepared for a mother to have skin-to-skin contact with her newborn?
10	Do you consider the current facilities adequate for extending skin-to-skin contact to all eligible women following caesarean section, and how would the routine introduction of this practice influence your attitude towards it?

**Table 2:** Sociodemographic and occupational characteristics of the participants (*n* = 40).

Code	Age	Gender	Civil Status	Children	Household Size	Occupation	Employment Status	Workplace
E1	29	Woman	Single	0	2	Anaesthetist	Casual worker	Operating theatre
E2	47	Woman	Married	3	5	Gynaecologist/obstetrician	Permanent contract	Maternity Delivery room High-risk unit
E3	63	Woman	Married	2	4	Midwife	Permanent contract	Delivery room
E4	28	Man	Single	0	1	Anaesthetist	Resident physician	Recovery room
E5	59	Woman	Married	3	5	Gynaecologist/obstetrician	Permanent contract	High-risk unit
E6	25	Woman	Single	0	1	Neonatologist	Resident physician	Delivery room
E7	64	Woman	Single	0	1	Midwife	Permanent contract	Delivery room
E8	61	Woman	Married	3	5	Nurse	Permanent contract	PACU
E9	42	Man	Married	2	4	Nurse	Interim contract	PACU
E10	46	Woman	Married	1	3	Nursing assistant	Permanent contract	PACU
E11	50	Man	Married	4	6	Nursing assistant	Interim contract	PACU
E12	28	Man	Single	0	2	Anaesthetist	Temporary contract	Operating theatre
E13	51	Woman	Married	2	4	Gynaecologist/obstetrician	Permanent contract	Maternity Delivery room High-risk unit
E14	63	Woman	Married	2	4	Midwife	Permanent contract	Delivery room
E15	29	Man	Single	1	3	Anaesthetist	Resident physician	PACU/Operating theatre
E16	27	Woman	Married	0	2	Gynaecologist/obstetrician	Temporary contract	High-risk unit
E17	25	Woman	Single	0	1	Neonatologist	Resident physician	Delivery room
E18	64	Woman	Single	0	1	Midwife	Permanent contract	Delivery room
E19	58	Woman	Married	3	5	Nurse	Permanent contract	PACU
E20	42	Woman	Married	2	4	Nurse	Interim contract	PACU
E21	53	Woman	Married	4	6	Nursing assistant	Permanent contract	PACU
E22	50	Man	Married	1	3	Nursing assistant	Permanent contract	PACU
E23	29	Man	Single	0	1	Anaesthetist	Temporary contract	Operating theatre
E24	51	Woman	Married	2	4	Gynaecologist/obstetrician	Permanent contract	Maternity Delivery room High-risk unit
E25	61	Woman	Married	2	4	Midwife	Permanent contract	Delivery room
E26	28	Man	Single	0	2	Anaesthetist	Resident physician	PACU/Operating theatre
E27	56	Woman	Married	3	5	Nurse	Permanent contract	PACU
E28	25	Woman	Single	2	4	Neonatologist	Resident physician	Delivery room
E29	38	Woman	Single	0	1	Midwife	Interim contract	Delivery room
E30	61	Man	Married	3	5	Nurse	Permanent contract	PACU
E31	39	Woman	Single	0	1	Nurse	Interim worker	PACU
E32	53	Woman	Married	1	3	Nursing assistant	Permanent contract	PACU
E33	50	Man	Married	4	6	Nursing assistant	Interim contract	PACU
E34	32	Man	Single	0	1	Anaesthetist	Temporary contract	Operating theatre
E35	54	Woman	Married	2	4	Gynaecologist/obstetrician	Permanent contract	Maternity Delivery room High-risk unit
E36	63	Woman	Married	2	4	Midwife	Permanent contract	Delivery room
E37	25	Man	Single	0	2	Anaesthetist	Resident physician	PACU/Operating theatre
E38	37	Woman	Married	0	2	Nurse	Interim contract	PACU
E39	46	Man	Married	0	2	Neonatologist	Permanent contract	Delivery room
E40	61	Woman	Single	0	1	Midwife	Permanent contract	Delivery room

## Data Availability

The anonymised codebook and thematic analysis framework generated in this study are available from the corresponding author (mebautista@utpl.edu.ec) upon reasonable request. Interview transcripts cannot be shared publicly due to ethical restrictions protecting participant confidentiality and the conditions of approval established by the Andalusian Research Ethics Committee. They may, however, be made available to authorised researchers under a formal data-sharing agreement.

## Supplementary Materials

The following supporting information can be downloaded at: https://www.mdpi.com/article/10.3390/healthcare14162650/s1, COREQ checklist.

## Abbreviations

SSC: skin-to-skin contact; NB: newborn; COREQ: Consolidated Criteria for Reporting Qualitative Research; SIDS: sudden infant death syndrome; DOHaD: Developmental Origins of Health and Disease; PACU: post-anaesthesia care unit; HCA: healthcare assistant; CFIR: Consolidated Framework for Implementation Research; TDF: Theoretical Domains Framework.
