# Supplementary material for: Barriers and Facilitators to Skin-to-Skin Contact After Caesarean Section: A Qualitative Study
Source: Healthcare (Basel). 2026 Aug 21;14(16):2650. doi: 10.3390/healthcare14162650 (PMC13512467; doi:10.3390/healthcare14162650)
Supplement: Supplementary file 1 [file healthcare-14-02650-s001.zip › healthcare-4446913-supplementary.pdf]

Supplementary Material: COREQ (32-Item) Checklist

Consolidated Criteria for Reporting Qualitative Research (COREQ): A 32-item checklist for interviews and focus groups.

No.	Item	Guide Questions/Description	Reported on Page/Section / Details from Manuscript
**DOMAIN 1: Research team and reflexivity**			
**Personal Characteristics**			
1	**Interviewer/facilitator**	Which author/s conducted the interview or focus group?	**Section 2.3:** Interviews were conducted by two members of the research team experienced in qualitative methodology.
2	**Credentials**	What were the researcher's credentials? (e.g. PhD, MD)	**Title \ Section 2.7:** Research team members hold credentials across Nursing, Medicine, and Health Sciences (PhD, RN, MD).
3	**Occupation**	What was their occupation at the time of the study?	**Title \ Section 2.7:** University researchers and clinical supervisors in Nursing and Health Sciences.
4	**Gender**	Was the researcher male or female?	**Title / Author list:** Both male (e.g., J.M.P.-J.) and female (e.g., R.d.D.-C., E.B.V.) researchers composed the study team.
5	**Experience and training**	What experience or training did the researcher have?	**Section 2.3 \ 2.7:** Experienced in qualitative research methodology, thematic analysis, and phenomenological designs.
**Relationship with participants**			
6	**Relationship established**	Was a relationship established prior to study commencement?	**Section 2.3 \ 2.4:** Interviewers were external to the clinical units to minimise hierarchical influence; no prior therapeutic or supervisory relationship was established.
7	**Participant knowledge of interviewer**	What did the participants know about the researcher?	**Section 2.4 \ 2.7:** Participants were provided with a written information sheet outlining the

No.	Item	Guide Questions/Description	Reported on Page/Section / Details from Manuscript
			study objectives, academic nature of the research, and confidentiality assurances.
8	**Interviewer characteristics**	What characteristics were reported about the interviewer/facilitator?	**Section 2.7:** Reflexivity is explicitly addressed; PI holds a clinical/managerial role, so external interviewers introduced the study as an exploration of practice rather than an evaluation.
**DOMAIN 2: Study design**			
**Theoretical framework**			
9	**Methodological orientation and Theory**	What methodological orientation was used to underpin the study?	**Abstract \ Section 2.1:** Descriptive qualitative exploratory design following a phenomenological approach and thematic analysis.
**Participant selection**			
10	**Sampling**	How were participants selected? (e.g. purposive, convenient)	**Section 2.3 \ 2.4:** Purposive sampling strategy targeting multidisciplinary healthcare professionals involved in the caesarean section care pathway.
11	**Method of approach**	How were participants approached? (e.g. face-to-face, phone)	**Section 2.4:** Approached directly at the hospital during staff changeovers (9:00 am–10:20 am).
12	**Sample size**	How many participants were in the study?	**Section 2.4 \ 3.1:** n = 40 healthcare professionals completed the study (from 52 approached).
13	**Non-participation**	How many people refused to participate or dropped out? Reasons?	**Section 2.4:** 12 non-participants (76.9% response rate): 8 declined due to lack of time/disinterest, and 4 were excluded due to scheduling conflicts.

No.	Item	Guide Questions/Description	Reported on Page/Section / Details from Manuscript
**Setting**			
14	**Setting of data collection**	Where was the data collected?	**Section 2.2 \ 2.4:** Spanish public tertiary university hospital (Andalusia); private rooms chosen by participants within the hospital to ensure privacy.
15	**Presence of non-participants**	Was anyone else present besides the participants and researchers?	**Section 2.4:** None; interviews took place in private locations free from interruption to guarantee confidentiality.
16	**Description of sample**	What are the important characteristics of the sample?	**Section 3.1 \ Table 2:** Detailed breakdown of *n* = 40 participants across 7 professional categories, including age, gender, contract type, and workplace.
**Data collection**			
17	**Interview guide**	Were questions, prompts, guides provided by the authors? Was it pilot tested?	**Section 2.5 \ Table 1:** Semi-structured interview guide with open-ended questions covering sociodemographic traits, SSC knowledge, training, interest, benefits, and infrastructure.
18	**Repeat interviews**	Were repeat interviews carried out?	**Section 2.3 \ 2.4:** No; single semi-structured individual interviews lasting approximately 30 minutes.
19	**Audio/visual recording**	Did the research use audio or visual recording to collect the data?	**Section 2.6:** Interviews were audio-recorded and transcribed verbatim for semantic and pragmatic qualitative analysis.
20	**Field notes**	Were field notes made during and/or after the interview or focus group?	**Section 2.6:** Field notes and divergence flags were recorded during data generation and coding.
21	**Duration**	What was the duration of the interviews or focus group?	**Section 2.4:** Approximately 30 minutes per interview, with additional time offered as needed.

No.	Item	Guide Questions/Description	Reported on Page/Section / Details from Manuscript
22	**Data saturation**	Was data saturation discussed?	**Section 2.3:** Saturation was assessed iteratively; recruitment ceased at 40 interviews when no new codes emerged across three consecutive interviews.
23	**Transcript returned**	Were transcripts returned to participants for comment and/or correction?	**Section 2.7:** Member-checking was conducted with selected participants to support credibility.
**DOMAIN 3: Analysis and findings**			
**Data analysis**			
24	**Number of data coders**	How many data coders coded the data?	**Section 2.6:** Two independent coders performed the analysis using MAXQDA 2022 software.
25	**Description of the coding tree**	Did authors provide a coding tree?	**Section 2.6 \ Figure 1:** Coding framework with 4 overarching categories and detailed sub-categories provided.
26	**Derivation of themes**	Were themes identified a priori or from the data?	**Section 2.6:** Mixed deductive-inductive thematic coding: initial codebook derived deductively from research questions and expanded inductively as new data emerged.
27	**Software**	What software, if any, was used to manage the data?	**Section 2.6:** MAXQDA 2022 software.
28	**Participant checking**	Did participants provide feedback on the findings?	**Section 2.7:** Credibility supported via member-checking with selected participants.
**Reporting**			
29	**Quotations presented**	Were participant quotations presented to illustrate the themes / findings? Was each quotation identified?	**Section 3.2–3.7:** Verbatim quotations presented throughout, identified by participant age, gender, and professional role (e.g., *Woman, 63 years old, midwife*).

No.	Item	Guide Questions/Description	Reported on Page/Section / Details from Manuscript
30	**Data and findings consistent**	Was there consistency between the data presented and the findings?	**Section 3 \ 4:** Yes; findings, negative case analysis, and verbatim quotes are fully integrated and cross-referenced with discussion themes.
31	**Clarity of major themes**	Were major themes clearly presented in the findings?	**Section 3.2–3.7 \ Figure 1:** Major themes structured logically across 4 main domains: Knowledge/Training, Acceptance/Interest, Benefits/Breastfeeding, and Infrastructure/Staffing Barriers.
32	**Clarity of minor themes**	Is there a description of diverse cases or discussion of minor themes?	**Section 2.6 \ 4.1:** Negative case analysis explicitly conducted; divergent viewpoints, age-related resistance differences, and professional group variations reported explicitly.
